# From data silos to integrated diagnosis: multimodal machine learning in glioblastoma

**DOI:** 10.3389/fonc.2026.1912365

**Published:** 2026-08-24

**Authors:** Amin Zadeh-Shirazi, Bryan W. Day, Hui K. Gan, Guillermo A. Gomez

**Affiliations:** 1 Centre for Cancer Biology, Adelaide University, Adelaide, SA, Australia; 2 Department of Computer Engineering, Faculty of Engineering, Khayyam University, Mashhad, Iran; 3 Sid Faithfull Brain Cancer Laboratory, QIMR Berghofer, Brisbane, QLD, Australia; 4 School of Biomedical Sciences, The University of Queensland, Brisbane, QLD, Australia; 5 Cancer Therapies and Biology Group, Centre of Research Excellence in Brain Tumours, Olivia Newton-John Cancer Wellness and Research Centre, Austin Hospital, Heidelberg, Melbourne, VIC, Australia; 6 La Trobe University School of Cancer Medicine, and Department of Medicine, University of Melbourne, Heidelberg, Melbourne, VIC, Australia

**Keywords:** artificial intelligence, clinical translation, computational pathology, glioblastoma, multimodal machine learning, radiogenomics, survival analysis, transformers

## Abstract

Effective glioblastoma care requires integrating multiparametric longitudinal MRI with histopathology, molecular profiling, and clinical records documenting surgery, radiotherapy, chemotherapy, and supportive treatments. In routine practice, however, these data streams are often evaluated separately rather than jointly, which can delay molecularly informed stratification, limit reproducibility across centers, and complicate interpretation of post-treatment imaging changes. Multimodal machine learning (MML) provides a framework for clinical decision support by integrating diverse patient data across the course of care, from symptom presentation through diagnosis to treatment decisions. By combining MRI, whole-slide pathology, molecular and methylation profiling, and treatment timelines derived from electronic health records, MML models can capture disease characteristics over time across biological scales through representation learning and multimodal fusion. Importantly, these approaches can incorporate uncertainty through model calibration and confidence-aware predictions. When rigorously developed and validated, MML models may generate clinically relevant outputs, including integrated diagnosis, molecular classification, individualized survival estimates, probabilistic discrimination between tumor progression and pseudo-progression, and stratification for clinical trial eligibility. In this Mini Review, we summarize recent advances and emerging translational evidence for clinically oriented MML in glioblastoma, with particular emphasis on MRI-centered systems that integrate imaging with pathology, selected molecular measurements, and longitudinal clinical context. We also outline key methodological and practical considerations, including dataset curation, leakage control, external validation, calibration, and post-deployment monitoring—required to support safe, robust, and generalizable implementation of MML approaches in neuro-oncology practice.

## Graphical Abstract

**Figure:**
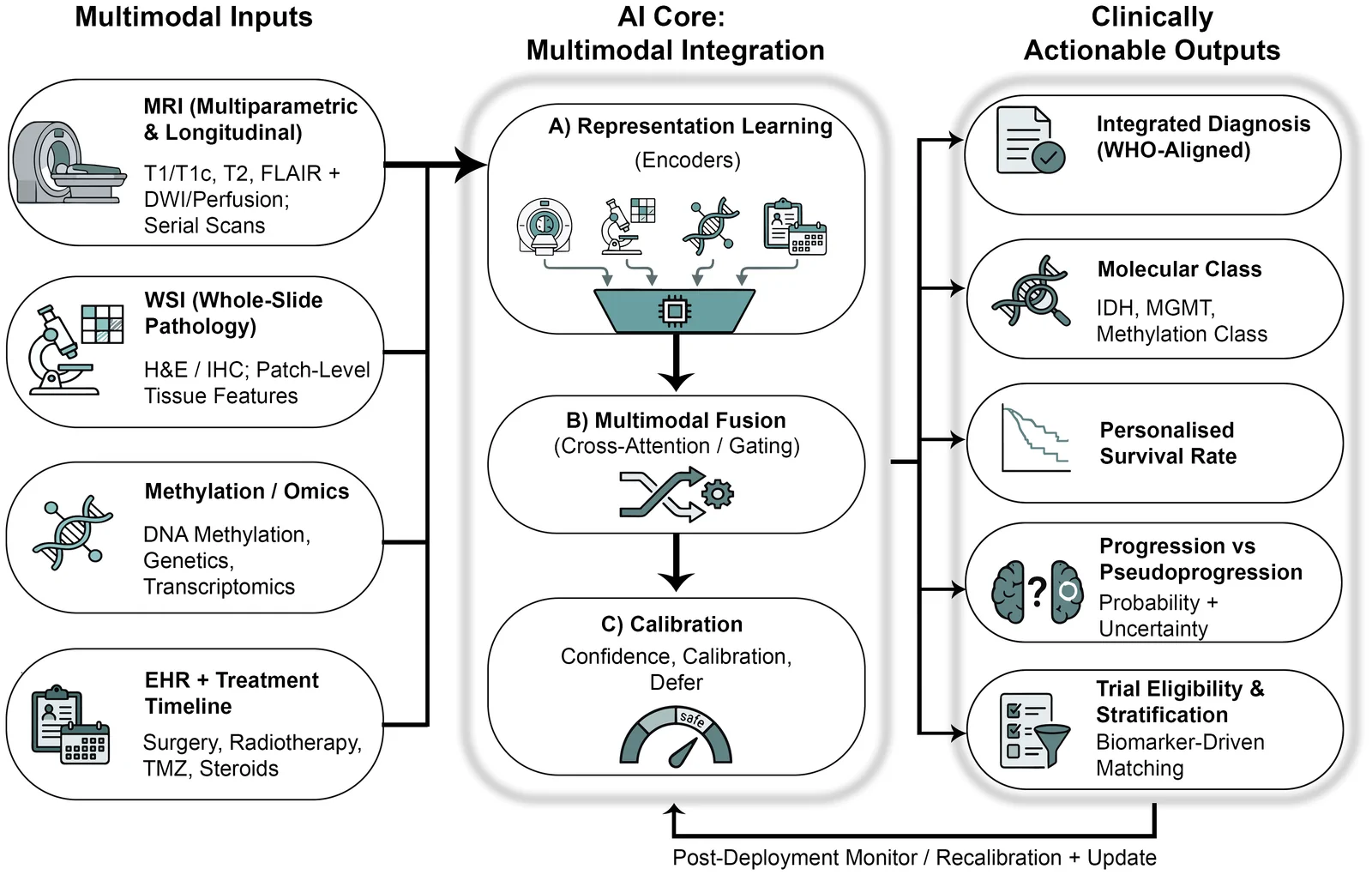
A multimodal machine learning framework for glioblastoma. Schematic overview of a multimodal pipeline integrating longitudinal multiparametric MRI, whole-slide pathology, methylation/omics, and electronic health record (EHR) treatment timelines within an artificial intelligence (AI) core comprising representation learning encoders, multimodal fusion, and uncertainty calibration for confidence-aware predictions. Multimodal machine learning models can generate clinically actionable outputs, including WHO-aligned integrated diagnoses, molecular classifications (e.g., IDH, MGMT, or methylation class), personalized survival estimates, discrimination between progression and pseudo-progression, and trial-eligibility stratification. A feedback loop enables post-deployment monitoring for drift, bias, and performance changes, supporting recalibration and model updates as clinical practice evolves.

## Highlights

- MML models are most reliable when trained and validated using integrated patient data—combining MRI, pathology, and molecular information—rather than relying on a single modality such as imaging alone.
- Effective MML models must robustly combine different data types, while accounting for missing information and changes over time, including treatment events and longitudinal follow-up.
- Time-aware MML models use only the data available at each clinical timepoint, mirroring real clinical workflows where imaging, pathology, molecular results, and clinical information become available at different times, hence reducing bias and supporting more consistent clinical decision-making.
- To be clinically useful, MML models should provide calibrated risk estimates, clear uncertainty measures, and transparent explanations that fit seamlessly within existing clinical workflows.
- Successful clinical translation of MML models requires stepwise validation, including external validation, prospective “shadow-mode” testing in real time without influencing care, and ultimately interventional clinical trials with transparent reporting.

## Introduction

Precision oncology increasingly relies on heterogeneous, complementary data streams, radiology, digital pathology, molecular profiling, and clinical variables that capture distinct aspects of tumor biology and the patient’s wellness state. Multimodal machine learning (MML) has therefore become a key methodological direction because it enables a multi-parametric model to fuse cross-scale signals, rather than relying on a single modality that provides an incomplete, biased, or context-dependent clinical picture. A prominent example is the pan-cancer integration of whole-slide histology with molecular profiles, an approach that has been deployed across 14 tumor types to predict outcomes and discover prognostic correlates (1). The pan-cancer effort illustrates the value of joint modeling beyond unimodal baselines (1), although there are still persistent challenges, particularly relating to data heterogeneity and integration complexity (2), as well as data incompleteness and modality imbalances that complicate this multimodal data integration (robust fusion) in the real-world (3).

Multimodal data integration offers the additional advantage of not being agnostic to the data it integrates and not being limited to imaging or molecular profiles. Indeed, recent studies have demonstrated that multimodal approaches can be clinically meaningful and advantageous when they combine modalities that reflect different “views” of the disease. For example, integrating radiology and histopathology has been shown to improve prognostic modeling. This was demonstrated in a head-and-neck cancer study, showing that combined approaches outperformed unimodal models. This supports the general principle that cross-modal fusion can strengthen outcome prediction when the modalities encode complementary tumor phenotypes (4). In parallel, multimodal machine learning prognosis frameworks continue to evolve toward integration of structured clinical signals, reflecting the rapid adoption and broader push to align AI outputs with real clinical decision making in clinical oncology (5).

Advances in these approaches offer important opportunities to improve the diagnosis and treatment of central nervous system cancers, particularly glioblastoma, where clinical management has changed little over the past three decades. Prognosis and treatment planning for glioblastoma have been historically very challenging. Like many cancers, glioblastoma exhibits intra-tumor and inter-tumor heterogeneity in histopathological analysis, likely biased by sampling variability. These factors, together with differences in institutional workflows, pose challenges to consistent diagnosis and downstream analyses. In this regard, multimodal deep learning has been utilized to integrate histopathology with gene expression analysis for prognostication across adult and pediatric brain tumor cohorts, yielding improvements over single-modality models (6). In glioblastoma specifically, a multicenter study reported transformer-based multimodal survival prediction integrating multiparametric magnetic resonance imaging (MRI) with clinical and pathological information (including MGMT promoter methylation status), reflecting the field’s movement toward clinically grounded, multi-source risk modeling (7). Complementary evidence also supports the prognostic relevance of multimodal neuroimaging (including structural MRI and resting-state fMRI) for pretreatment survival stratification (8). As in many cancer types, translation remains constrained by practical realities, and these constraints are particularly consequential in glioblastoma because response interpretation is time-dependent and confounded by therapy-related imaging changes. most notably missing or inconsistently acquired MRI series/protocols (e.g., T1, post-contrast T1, T2, FLAIR), which have motivated dedicated methodological work and surveys on learning under missing-modality conditions in brain tumor imaging (9). This problem is most evident in multi-center retrospective clinical cohorts and public neuroimaging repositories, where protocol completeness varies across sites and time points, highlighting the need to develop missing-modality learning methods.

Against this backdrop, our review of the literature has brought us to the summation that the next critical step for safe, robust, and generalizable implementation of MML in neuro-oncology practice is not only improved fusion (i.e. improved datasets and/or robust fusion protocols), but also improved *assurance at decision time*: ensuring that (i) model inputs are admissible at the time of clinical decision making (to prevent temporal leakage), (ii) evaluation reflects realistic deployment (train/validation/test partitioning, ideally patient-level with site-stratified and/or time-aware splits where relevant, plus external validation and robustness checks), and (iii) outputs are communicated in a way that supports safe action (uncertainty-aware flagging/deferral rather than overconfident point predictions). By explicitly organizing these safeguards into a practical assurance paradigm aligned with real neuro-oncology decision points, we present a framework that aims to bridge the gap between promising multimodal performance reports and credible real-world clinical translation.

Accordingly, the scope of this Mini Review is deliberately imaging-led rather than an exhaustive survey of multi-omics technologies. We focus on clinically oriented MML systems in which MRI provides the principal longitudinal phenotype and is integrated, when available, with pathology, molecular measurements, and EHR-derived treatment timelines. This scope is aligned with the article’s central translational question, how heterogeneous patient information can be used safely and credibly at a defined clinical decision time, rather than with an assay-by-assay comparison of molecular platforms.

## Data modalities in glioblastoma

**Figure 1:**
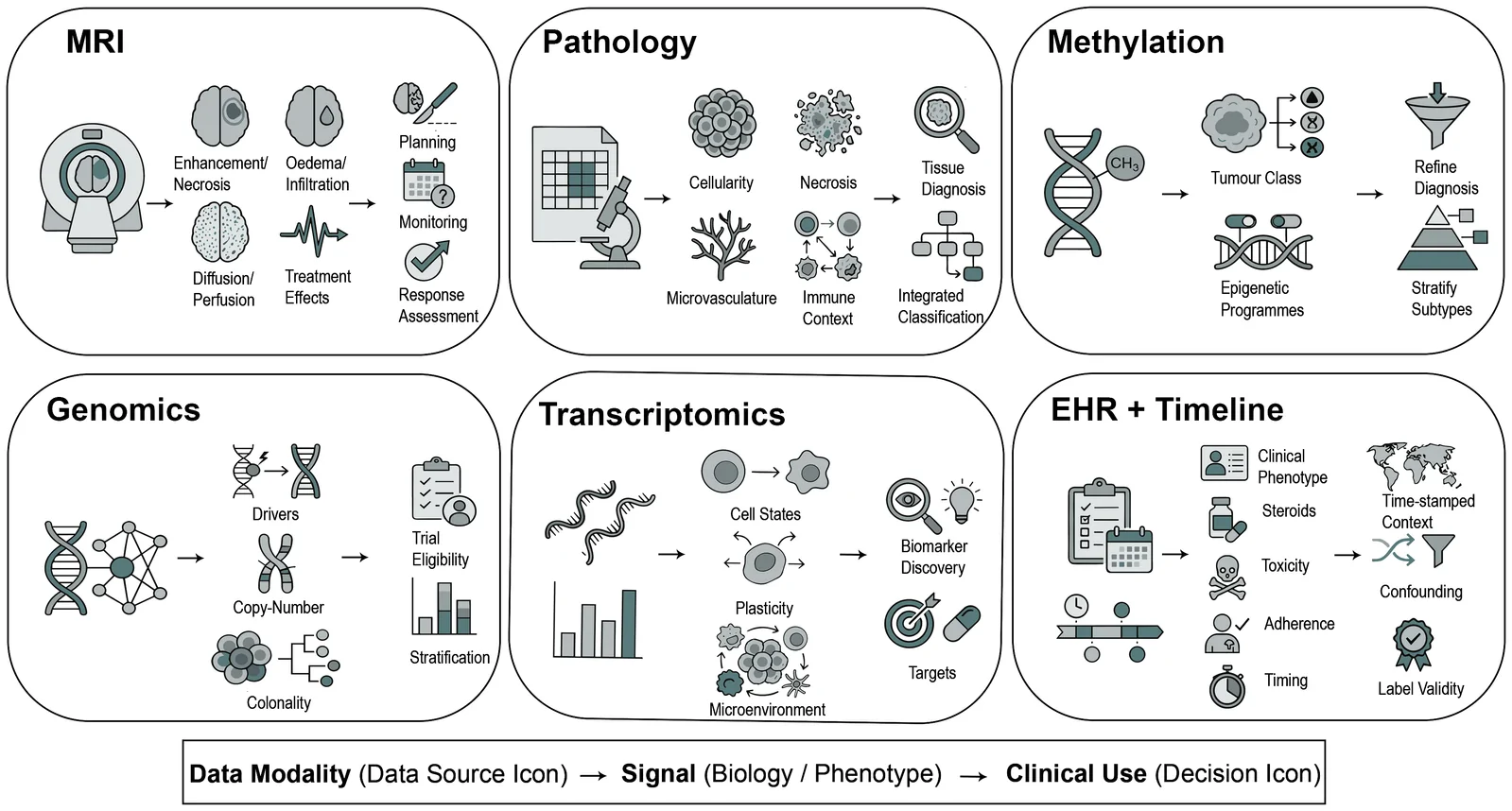
Principal glioblastoma data modalities and their clinical roles. Six panels summarize common evidence streams used in glioblastoma care, MRI, neuropathology, DNA methylation, genomics, transcriptomics, and EHR plus treatment timeline, organized as data source → biological/phenotypic signal → clinical use. The schematic emphasizes how different modalities resolve distinct layers of disease biology and how longitudinal clinical context (including treatment timing, steroids, toxicity, and adherence) is required to mitigate confounding and ensure label validity in predictive modeling.

Glioblastoma care relies on integrating evidence across spatial and temporal scales, from molecular alterations to tissue architecture, whole-brain imaging, and aligning these findings to the patient’s longitudinal treatment journey. **Figure 1** outlines a concise “signal-to-decision” roadmap across the main modalities used in contemporary glioblastoma diagnosis. MRI provides the dominant non-invasive, repeatable readout of macroscopic tumor phenotype over time, capturing enhancement patterns (blood–brain barrier disruption), necrosis, peri-tumoral edema/infiltration, diffusion/perfusion surrogates of cellularity and vascularity, and therapy-related imaging effects that complicate response interpretation. Standardized acquisition and reporting conventions are therefore essential if imaging-derived features are to generalize across scanners of different makes, centers, and trials, and if models are to remain valid under protocol drift and evolving clinical practice (10). Neurohistopathological analysis of resected or biopsied tumor tissue reveals tissue architecture and cellular morphology, enabling direct assessment of cellularity, mitotic activity, necrosis, microvascular proliferation, and the inflammatory microenvironment, features that have been further refined through the implementation of different spatial omics technologies (11). These are essential features that anchor diagnostic classification and provide a mechanistic context for molecular readouts. In modern practice, histopathology is interpreted within an integrated diagnostic framework that combines morphological and molecular pathology (i.e., TERT-promoter and IDH mutation status, MGMT methylation, and chromosomal changes like 7p+/10q- and IDH mutant status), underscoring the central role of pathology-derived signals in definitive diagnosis and downstream treatment intent (12). However, current neuropathological assessment is affected by observer bias and sampling variability, and computational pathology can improve its reproducibility by quantifying morphological patterns at scale, though it remains limited by the tissue sampled. More recently,DNA methylation profiling in molecular diagnostic laboratories provides a high-dimensional epigenetic fingerprint that enables robust tumor classification, refinement of diagnostically ambiguous cases, and stratification into biologically meaningful subgroups (13). In glioma diagnostics, methylation-based classification has demonstrated strong utility for resolving entities and for harmonizing classification across centers, making it a highly valuable modality for multimodal fusion when tissue is available and assay pipelines are standardized (14). Genomics (targeted panels, exomes, or broader profiling) captures driver alterations, copy-number changes, and clonal structure that inform prognostic biology, potential therapeutic vulnerabilities, and eligibility for biomarker-defined trials. Large-scale efforts defining the genomic landscape of glioblastoma illustrate both the richness of genomic signal and the need to interpret it in context, particularly where sampling bias and intratumor heterogeneity can decouple a biopsy-derived molecular snapshot from the evolving disease process (15). Transcriptomics adds a functional layer by capturing gene expression programs that reflect malignant cell states, plasticity, and microenvironmental interactions. Beyond genomics, methylation profiling, and transcriptomics, proteomic and metabolomic measurements provide additional molecular layers that capture functional consequences of genomic alterations and dynamic tumor states. Proteomics can characterize protein abundance, signaling pathway activation, post-translational regulation, and tumor–microenvironment interactions, whereas metabolomics reflects altered metabolic programs associated with proliferation, adaptation, and therapeutic response. In glioblastoma, these modalities have contributed to the identification of biologically relevant molecular subgroups and treatment-associated changes, although their integration into routine clinical workflows remains limited by technical standardization, tissue availability, and longitudinal sampling challenges. From a multimodal learning perspective, proteomic and metabolomic data represent complementary biological constraints that may enhance future models when harmonized with imaging, pathology, molecular profiling, and clinical timelines. Single-cell and integrative transcriptomic studies highlight that glioblastoma comprises multiple coexisting cellular programs that vary across space and time, motivating multimodal approaches that link molecular states to imaging phenotypes and clinical trajectories rather than assuming a single static tumor identity (16). Finally, Electronic Health Records (EHR) and Treatment Clinical Variables (for example, performance status proxies, steroid exposure, timing of surgery and radiotherapy, dose intensity, adherence, toxicity, and follow-up scheduling) provide the indispensable longitudinal context that determines which labels are clinically meaningful and whether a prediction task is well posed. For modeling, these variables are not merely covariates: they define time zero, establish temporality (train precedes test), and help prevent confounding and label leakage when outcomes are influenced by post-baseline decisions. A target-trial mindset is therefore critical when using routinely collected clinical data to build decision-support models intended to guide real-world strategy selection (17).

## Glioblastoma & multimodal machine learning

Unimodal modeling in glioblastoma is limited by coupled biological and measurement uncertainty. Tumor states are heterogeneous across space and evolve under therapy, so any single biopsy fragment or imaging timepoint is an incomplete snapshot (16, 18–20). In parallel, real-world measurement processes vary by scanner, protocol, and laboratory pipeline, creating site shift and appearance variation that can mislead models unless explicitly controlled (21, 22). These structural uncertainties motivate multimodal learning and, critically, decision-time assurance to prevent leakage and overconfident deployment in high-stakes settings (23). In practical terms, this complexity is why single-modality models often fail to generalize across centers and why prognosis and post-treatment imaging interpretation remain uncertain in routine care.

**Figure 2:**
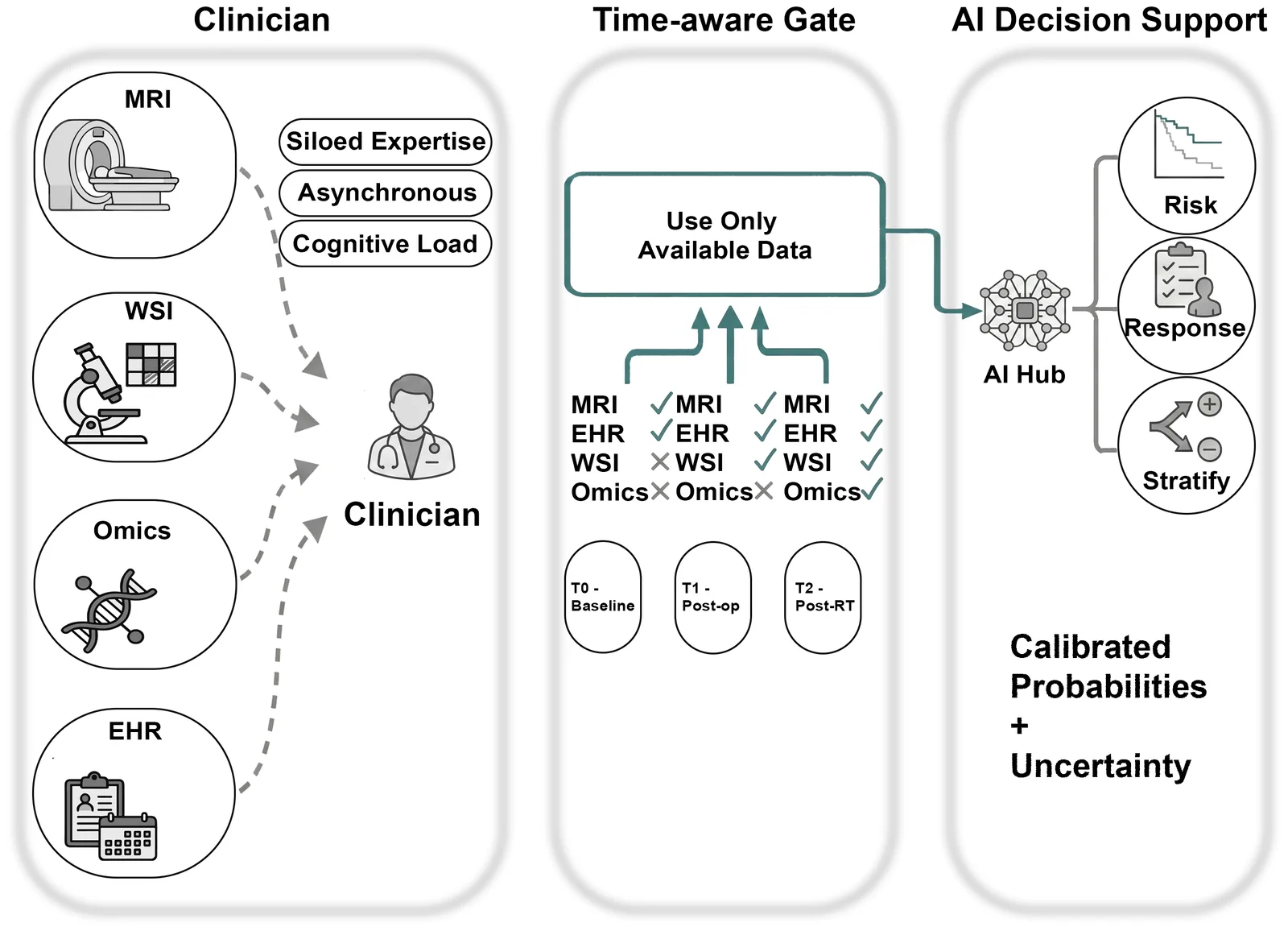
Time-aware multimodal integration for glioblastoma decision support. Left, Clinicians integrate heterogeneous data (MRI, whole-slide imaging, molecular/omics assays, and EHR context), but this process is limited by siloed expertise, asynchronous data availability, and cognitive load. Center, A time-aware admissibility gate restricts model inputs to data available at the decision time (t0 baseline; t1 post-operative; t2 post-radiotherapy), preventing the use of future information. Right, MML model outputs consist of calibrated probabilistic decision support for risk stratification, response assessment, and clinical stratification, while retaining uncertainty to guide deferral or additional data acquisition.

In this work, time-aware modeling means restricting predictors and labels to information available at the intended decision time and respecting the temporal order in splitting (training precedes testing; **Figure 2**). Leakage denotes any inadvertent use of information unavailable at deployment or of spuriously predictive cues (for example, site/protocol artifacts or post-treatment variables contaminating pre-treatment tasks). Uncertainty is the model’s quantified lack of confidence in a decision-relevant latent state (for example, progression versus pseudo-progression), and calibration is the agreement between predicted probabilities and observed frequencies. Shift refers to distributional changes between training and deployment (scanner/site/protocol or laboratory effects), and missingness-aware inference denotes methods that remain robust when modalities or time points are absent. Finally, confounding arises when treatment choices and timing influence both inputs and outcomes, producing non-causal associations that can inflate apparent performance.

From a methodological perspective, MML systems generally use modality-specific representation learners. In the studies considered here, MRI or whole-slide pathology is encoded using convolutional or transformer-based networks, whereas molecular and structured clinical variables are represented using dedicated neural feature encoders before fusion [1,6,7]. Fusion may occur at the early, intermediate or joint, or late stage, with attention, cross-attention, and gating mechanisms used to model interactions between modalities [1,6,7]. For prognostic tasks, published brain-tumor models have used Cox proportional-hazards outputs or nonlinear and nonproportional deep-survival models [6,7]. Missing imaging modalities may be addressed through image synthesis, latent-feature-space learning, correlation-based methods, knowledge distillation, or domain adaptation [9]. No single architecture is universally optimal; model selection should reflect modality alignment, cohort size, missingness, interpretability requirements, and the intended clinical decision. To ground the above-described mechanistic rationale framework in evidence, in **Table 1**, we included peer-reviewed studies reporting human glioblastoma cohorts that applied multimodal modeling with at least two distinct data modalities and evaluated a clinically relevant endpoint/task (for example, survival/prognosis, molecular classification, response assessment, or decision support). We excluded studies restricted to non-glioblastoma entities, non-human data, single-modality models, purely methodological papers without clinical endpoints, and studies lacking sufficient cohort and evaluation reporting. For each eligible study, we recorded the definition and implementation of “multimodal” (fusion stage: early/intermediate/late; and whether modalities were aligned at the patient/timepoint level or unaligned), together with translation-critical evaluation characteristics: validation level (internal/external/prospective), split protocol (patient-level; site-stratified; time-aware where relevant), calibration/uncertainty reporting —to allow critical assessment of robustness and leakage/bias risk, and whether unimodal baselines were provided to quantify incremental benefit. Moreover, to clarify how retrospective performance relates to clinical readiness, we organized the evaluation into a simple translation ladder: internal validation (within-cohort resampling or held-out splits) and external validation (testing on independent institutions or acquisition distributions). In this framework, claims of clinical usefulness should be weighted by the highest achieved level, because glioblastoma datasets are particularly susceptible to site shift, treatment-time confounding, and selection bias. Altogether, this structure allows **Table 1** to be interpreted as evidence not only of performance, but of robustness and clinical credibility under realistic deployment risks.

**Table 1:** Representative clinically evaluated multimodal glioblastoma studies and translation-relevant evaluation characteristics.

Table 1A Study characteristics and primary performance.							
Ref #	Year	Datasets used	Sample No.	Modalities	Task	Performance	Multimodal vs best unimodal (Δ metric; explicit)
(7)	2024	UPenn-GBM; UCSF-PDGM; RHUH-GBM (3 public cohorts)	Total 780 (GBM 780): UPenn 378; UCSF 366; RHUH 36	MRI; EHR + Timeline; DNA methylation (MGMT status)	Overall survival prediction (time-to-event)	Time-dependent concordance (Cdt) 0.672 (UCSF external, setup 1)	Δ Cdt = +0.094 (0.672 vs 0.578 imaging-only; UCSF external)
(31)	2025	Burdenko GBM progression cohort; UKER GlioCMV progression cohort; SSL pretraining: BraTS2021, UPenn-GBM, UCSF-PDGM	79 (GBM 79): Burdenko 59; UKER 20	MRI; EHR + Timeline	Post-radiotherapy progression vs pseudoprogression classification	AUC 0.753 (external UKER test)	NR
(32)	2022	UPenn GBM cohort (single institution)	516 (GBM 516): discovery 404; replication 112	MRI; EHR + Timeline; Genomics; DNA methylation	Overall survival prediction (Cox proportional hazards)	C-index 0.75 (multimodal Cox; replication cohort)	Δ C-index = +0.10 (0.75 vs 0.65 clinical-only)
(33)	2019	TCIA/TCGA-GBM; McGill University Health Centre (MUHC)	200 (GBM 200; IDH1-wild-type): TCIA/TCGA 71; MUHC 129	MRI; EHR + Timeline; Genomics; Pathology	Survival-related outcome classification	AUC 0.7824 (integrative model; 10-fold CV/100–100 split)	NR
(34)	2022	Zhongnan Hospital (local); TCGA/TCIA	142 (GBM 142; IDH-wild-type): local 70; TCGA/TCIA 72	MRI; EHR + Timeline	Preoperative survival prediction; nomogram for 1- and 2-year survival	C-index 0.74–0.86 (radiomics and radiomics + clinical models)	NR
(35)	2024	BraTS 2020; STORM_GLIO (multicenter retrospective)	289 (GBM = 289)	MRI; EHR + Timeline (age)	Overall survival prediction (time-to-event risk stratification)	C-index 0.69 (95% CI 0.62–0.75) on validation; iAUC at 11 months 0.81	Δ iAUC at 11m = +0.14 (0.81 vs 0.67 age-only; validation)
(36)	2024	Single-institution development + external validation cohort	156 (GBM 156): development 116; external test 40	MRI; EHR + Timeline (age); DNA methylation (MGMT status)	Binary survival endpoint: survival ≥18 months vs <18 months	AUC 0.89 (external test; multimodal model)	Δ AUC = +0.18 (0.89 vs 0.71 radiomics-only; external test)
(6)	2023	TCGA adult glioma cohort (TCGA-GBM+TCGA-LGG); external CPTAC-GBM	TCGA: 783 (GBM 357) + CPTAC: 97 (GBM 97)	Pathology (WSI); Transcriptomics	Survival risk prediction (Cox-PH)	Composite score (CS) 0.710 on CPTAC; IBS 0.174 (early fusion)	Δ CS = +0.031 (0.710 vs 0.679 best unimodal RNA; CPTAC)
Table 1B Fusion strategy and rigor (translation-relevant safeguards).							
Ref #	Fusion stage (early/intermediate/late)		Aligned	Validation level (internal/external/prospective)	Split protocol (patient-level; site-stratified; time-aware)	Calibration/uncertainty reported (Y/NR; metric if Y)	
(7)	Intermediate		Yes	External (multi-site independent cohorts)	Two training setups with cross-site testing across UPenn/UCSF/RHUH (patient-level)	Y; integrated Brier score (IBS)	
(31)	Intermediate		Yes	External (UKER)	Train/validation on Burdenko; external test on UKER (patient-level)	NR	
(32)	Early		Yes	Internal (discovery/replication)	Patient-level split: discovery (n=404) vs replication (n=112)	NR	
(33)	Early		Yes	Internal	10-fold cross-validation; random train/test split (100/100)	NR	
(34)	Early		Yes	External (TCGA/TCIA test cohort)	Patient-level split: training 100 (70 local + 30 TCGA/TCIA); test 42 TCGA/TCIA	Y; calibration curves (no scalar metric reported)	
(35)	Early		Yes	Internal	Random 80/20 patient-level split; repeated 3-fold CV (33 reps) on training; bootstrap	Y; calibration (plots; no scalar metric)	
(36)	Early		Yes	External	External hold-out institution (n=40)	NR	
(6)	Early		Yes	External	External test on CPTAC-GBM	Y; integrated Brier score (IBS 0.174 on CPTAC)	

PsP, pseudoprogression; TP, true progression; AUC, area under the ROC curve; C-index, concordance index; IBS, integrated Brier score; Cdt, time-dependent concordance; CS, composite score; NR, not reported.

Scope note: **Table 1** focuses on clinically oriented MML studies evaluating patient-level clinical endpoints and translation-relevant safeguards. It is not intended as a comprehensive catalogue of standalone molecular profiling or multi-omics studies in glioblastoma.

The predominance of MRI-containing studies in **Table 1**, seven of the eight representative studies, is therefore a characteristic of the defined, clinically oriented evidence base rather than an assertion that molecular data are secondary. Molecular measurements provide complementary and non-interchangeable biological information. Genomic and DNA-methylation assays can provide comparatively stable signals for integrated diagnosis, tumor classification, and biomarker stratification, whereas transcriptomic, proteomic, and metabolomic measurements can capture more dynamic cellular programs, pathway activity, and treatment-associated states. Their principal advantages include biological specificity, mechanistic resolution, and the potential to refine biomarker-defined stratification. Their principal limitations for clinically oriented MML include dependence on invasive tissue acquisition, regional sampling bias, limited longitudinal repeat sampling, high dimensionality relative to cohort size, assay- and batch-dependent variation, and delayed or incomplete availability at the intended decision point. These characteristics make molecular data complementary to, rather than interchangeable with, longitudinal imaging and reinforce the need for patient- and timepoint-level alignment, missingness-aware inference, leakage-resistant preprocessing, and independent validation. A comprehensive modality-by-modality appraisal of molecular omics technologies would require a separate review question, literature-selection strategy, and evidence framework and is therefore outside the focused scope of this Mini Review.

Studies in **Table 1** reflect two patterns that support the view that multimodal AI can reduce avoidable decision uncertainty when appropriately designed. First, multimodal systems are predominantly applied to tasks that are intrinsically underdetermined from any single modality, most notably prognosis/survival modeling and post-treatment response classification, where complementary evidence is required to constrain interpretation. Second, in the subset of studies that explicitly report unimodal comparators, multimodal integration yields a measurable improvement over the best reported single-modality baseline (captured in the *“Δ metric; explicit”* column), providing direct empirical support for incremental value beyond unimodal modeling. Together, these MML-studies support three takeaways (1). Multimodal systems are most often applied where single modalities are underdetermined, such as prognosis and post-treatment response, so complementary evidence is required (2). Reported gains are most credible when supported by leakage-resistant evaluation (patient-level splits, time-aware design for longitudinal tasks, and external validation), because site/protocol cues and post-treatment variables can inflate apparent performance (3). Calibration and uncertainty reporting remain inconsistent, indicating a clear translational gap: clinically actionable systems should report calibrated probabilities and define uncertainty-guided deferral/flagging policies.

**Figure 3:**
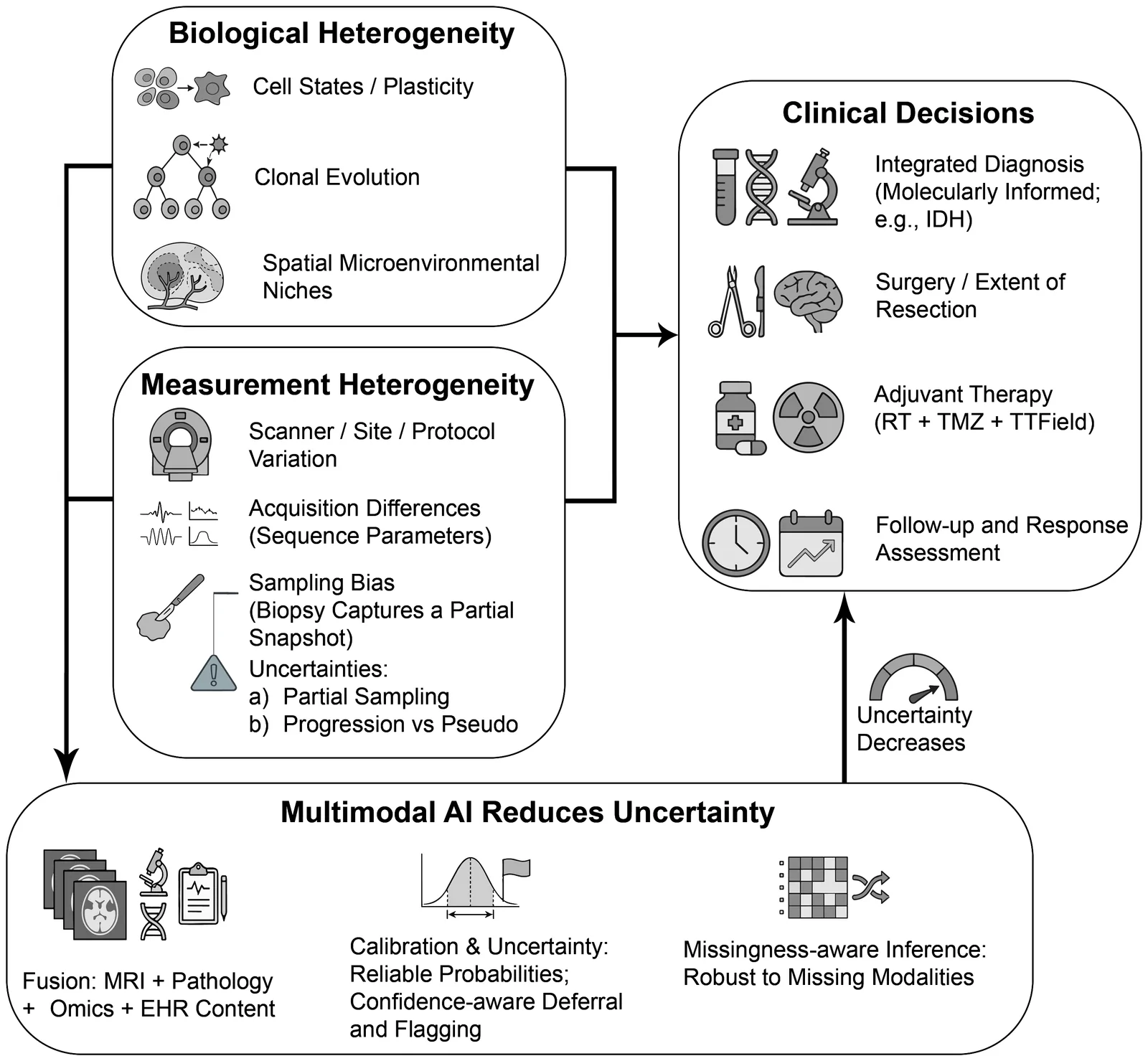
Clinical problem map motivating MML models for glioblastoma. Schematic linking biological heterogeneity (cell-state plasticity, clonal evolution, spatial niches) and measurement heterogeneity (scanner/site variation and sampling bias) to key clinical decisions in glioblastoma, including diagnosis, extent of resection, therapy selection, and response assessment. Major sources of uncertainty—such as partial tissue sampling and ambiguous post-treatment imaging (progression vs. pseudo-progression)—are highlighted. The lower panel illustrates how multimodal machine learning (MML) can reduce uncertainty by integrating complementary data, producing calibrated predictions, and handling missing data, while also emphasizing the need for time-aware evaluation and external validation to ensure reliable clinical translation.

**Figure 4:**
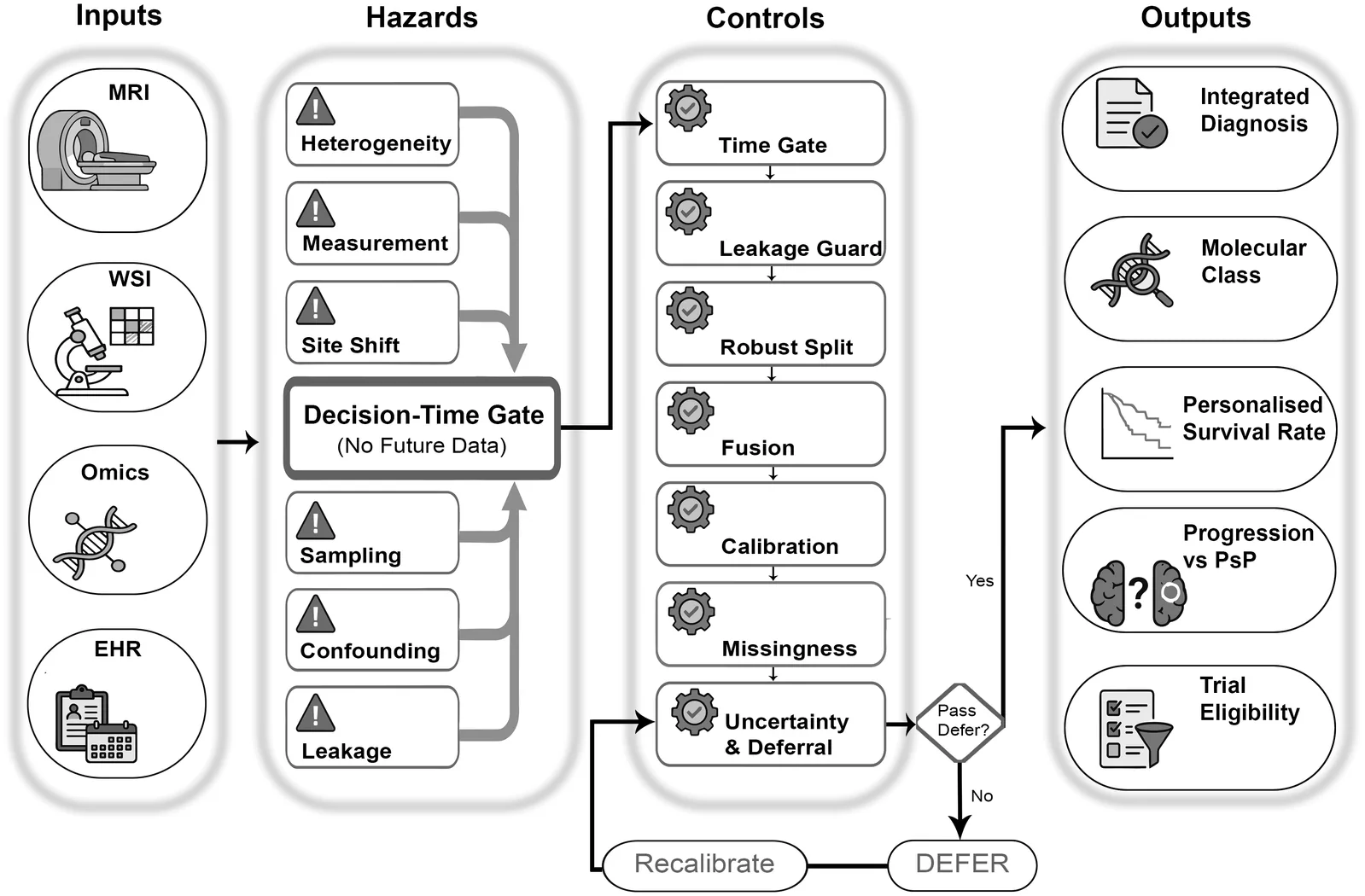
Decision-anchored assurance paradigm for multimodal AI in glioblastoma. Serial imaging, tissue pathology, molecular assays, and EHR data provide complementary inputs but are affected by biological and measurement heterogeneity, partial sampling, and potential data leakage. A decision-time admissibility gate ensures that only information available at the clinical time point is used. An assurance layer—including leakage control, robust evaluation, multimodal fusion, calibration, missingness handling, and uncertainty-guided deferral—determines whether outputs are released (PASS) or deferred for further evidence (DEFER). MRI, magnetic resonance imaging; WSI, whole-slide imaging; EHR, electronic health record; PsP, pseudo progression.

**Figures 1–4** are intended as complementary, high-level schematics: **Figure 1** summarizes the principal biological data domains, **Figure 2** their decision-time availability, **Figure 3** the biological and clinical uncertainties motivating multimodal integration, and **Figure 4** the methodological assurance controls required for credible deployment.

## Clinical assurance framework for MML in glioblastoma

Multimodal AI for glioblastoma is most usefully framed as decision-anchored inference under uncertainty, governed by an explicit assurance layer rather than treated as a generic predictive pipeline. As summarized in **Figure 3**, model failure and clinical ambiguity arise from interacting hazards: *biological heterogeneity* (spatially and temporally varying malignant programs and niches), *measurement heterogeneity and site shift* (scanner/protocol and laboratory variability), *sampling limitations* (biopsy or resection capturing a partial snapshot), and *temporal confounding* (treatment influences both observed measurements and outcomes). These hazards also amplify the risk of *leakage*, whereby models exploit information that would not be available at the intended clinical decision point (for example, post-treatment variables or site/protocol proxies), inflating apparent performance while undermining deployability.

These controls are consistent with established healthcare-AI guidance, including TRIPOD+AI for transparent reporting of prediction-model studies (24), PROBAST+AI for assessment of quality, risk of bias, and applicability (25), DECIDE-AI for early-stage clinical evaluation of AI-based decision-support systems (26), and FUTURE-AI for trustworthy development, deployment, and lifecycle monitoring (27). Our proposed framework does not claim these individual principles as novel; rather, it organizes them around the specific requirements of multimodal glioblastoma care, including asynchronous data availability, treatment-dependent measurements, decision-time admissibility, and uncertainty-guided deferral.

To address these hazards, we propose a minimal set of controls that define a clinically credible multimodal system (**Figure 4**). A central *decision-time gate* enforces time-aware admissibility by restricting all inputs to information available at the decision point and excluding variables derived from the future. Downstream, the assurance layer combines leakage guarding, robust splitting (patient-level and site-aware evaluation where feasible), and multimodal fusion to integrate complementary constraints across scales (phenotype, tissue architecture, molecular programs, and treatment context). Crucially, outputs are treated as calibrated probabilities with quantified uncertainty, enabling an explicit *PASS/DEFER* policy: high-confidence predictions proceed to clinically actionable outputs, whereas uncertain cases are deferred for additional evidence, review, or short-interval reassessment. Deferred cases also trigger a recalibration loop, supporting continual reliability amid distribution shifts and evolving clinical practice.

## Discussion

MML for glioblastoma is increasingly a viable route to more clinically credible decision-making and support because it addresses a core limitation of unimodal modeling: incomplete and heterogeneous observations of a multi-scale, treatment-perturbed disease process. Importantly, these apparent gains are not simply a function of “adding more data”; they are mediated by whether modeling choices respect the clinical decision context (time), mitigate bias and leakage, and produce calibrated, usable outputs under uncertainty.

Mechanistically, multimodal benefit is plausible because each modality constrains different layers of the same latent clinical state. MRI captures macroscopic phenotype and longitudinal change; whole-slide pathology provides tissue architecture and microenvironmental context; molecular assays constrain tumor class and programs; and EHR-derived treatment timelines encode exposures and timing that influence both measurements and labels. When integrated appropriately, these sources can serve as complementary constraints, reducing avoidable uncertainty that would otherwise arise when a model is forced to infer biological processes from a single, context-dependent snapshot. However, multimodal AI should not be interpreted as eliminating irreducible ambiguity: sampling limitations, non-identifiability, and therapy-induced imaging changes can preserve uncertainty even under ideal modeling. The clinically relevant promise is therefore twofold: (i) improved discrimination where modalities contribute non-redundant signal under rigorous evaluation, and (ii) improved reliability and safety when systems explicitly quantify residual uncertainty and act on it (for example, via deferral or flagged review).

Developments beyond glioblastoma also provide transferable methodological opportunities. Pan-cancer MML integrating histopathology with genomic and transcriptomic profiles has demonstrated the capacity of cross-modal representations to support prognostic modelling and discovery of outcome-associated features across tumor types (1). More recent pathology and vision–language foundation models, including CONCH, CHIEF, and MUSK, learn reusable representations from large multi-cancer image and text collections and can be adapted to diagnosis, biomarker prediction, and outcome modelling (28–30). In glioblastoma, such pretraining may reduce dependence on large disease-specific labelled cohorts and support integrated diagnosis, molecular-state prediction, and prognostic stratification. However, transfer from pan-cancer data should not be assumed to establish clinical utility: glioblastoma-specific adaptation, independent external validation, calibration, and assessment of domain shift and decision-time admissibility remain necessary before diagnostic or treatment-related use.

The predominance of patient-level aligned multimodal designs in **Table 1** reflects a practical truth: multimodal learning is most informative when measurements correspond to the same underlying patient state and decision point. In real-world workflows, however, modalities are frequently incomplete, asynchronous, or selectively acquired, making missingness a first-order design variable rather than a nuisance. This observation supports a shift from “multimodal as an ideal dataset property” to “multimodal as a deployment constraint”: robust systems must explicitly model patterns of missingness and availability and must clearly state which decision is supported when specific modalities are absent. Similarly, the choice of fusion stage (early, intermediate, late) should be justified in relation to clinical intent. Late fusion can be pragmatic for modular deployment, whereas intermediate (representation-level) fusion may better capture cross-modal dependencies; neither choice is inherently superior without clear alignment to the decision context, evaluation design, and available supervision.

Across the evidence base, credible translation requires a guardrails-first approach to design and reporting. At minimum, studies should (i) implement explicit leakage checks (excluding post-decision variables and site/protocol identifiers that trivially encode outcomes), (ii) enforce time-aware modelling and splitting for longitudinal or treatment-dependent tasks (training precedes testing in time; predictors restricted to information available at the intended decision point), (iii) evaluate robustness to site shift via site-stratified splits and/or external validation, (iv) report calibration and uncertainty (for example, calibration curves and a scalar metric such as Brier score or integrated Brier score, with a defined deferral/flagging rule where applicable), and (v) document and address missingness (how absent modalities/timepoints were handled, and whether availability patterns stratified performance). These requirements are not cosmetic: without them, reported improvements risk reflecting shortcut learning, confounding, or dataset-specific artifacts rather than a clinically meaningful signal. In this sense, evaluation is part of the model, because it defines what the model is permitted to learn and what performance claims can legitimately be generalized.

The literature summarized in **Table 1** suggests that the most persuasive claims are those supported by (at least) patient-level splitting, explicit external validation, and calibrated probabilistic outputs. Conversely, results derived solely from internal validation, especially where site effects are plausible and where longitudinal labels are treatment-dependent, should be interpreted as hypothesis-generating rather than deployment-ready. This distinction matters because glioblastoma care contains several high-stakes decision points (integrated diagnosis, adjuvant strategy, response interpretation, and trial eligibility) where false confidence can be harmful. The paradigm in **Figure 4** formalizes a practical stance on translation: multimodal AI should be deployed as decision support with admissibility constraints, not as an unconstrained predictor. Outputs should be released only when the system is within its validated domain, and calibrated uncertainty is acceptably low; otherwise, the correct action is deferral, additional evidence acquisition, or human review.

Despite rapid progress, several limitations remain consistent across studies. First, domain shift (scanner/protocol and laboratory variation) continues to threaten generalization, motivating harmonization, site-stratified evaluation, and pre-registered external testing. Second, time dependence is under-addressed for response tasks: many pipelines do not fully operationalize decision-time admissibility, leaving residual risk of temporal confounding and leakage. Third, uncertainty reporting is inconsistent, indicating a gap between methodological awareness and routine translational practice; calibration and uncertainty-guided deferral should become standard for clinically actionable endpoints. Finally, the field would benefit from clearer articulation of the clinical decision supported by each model, including the intended user, the thresholding/deferral policy, and the failure modes under which the model should not be used. Finally, multimodal glioblastoma AI is ready to move from isolated predictors to decision-time clinical systems, but real-world impact depends less on “bigger models” than on dependable integration: the right infrastructure (digital pathology where relevant, secure storage/compute, and PACS/LIS/EHR integration), sustained maintenance, and workflow redesign so outputs arrive when decisions are made. Deployment can scale center-by-center or via shared state/national platforms, provided governance supports privacy, security, auditability, and continuous monitoring for drift and bias, especially given that glioblastoma is a uniquely demanding stress test in which evidence arrives asynchronously and treatment perturbs response signals. To make gains meaningful, studies should link predictions to predefined clinical actions (e.g., trial triage, intensified monitoring, imaging interval changes) and report decision-analytic benefit, calibration, and uncertainty-aware deferral, while addressing environmental costs (efficient training, model reuse) and equity (robust multi-site performance with “graceful degradation” when modalities are missing). The clearest path to routine use is prospective shadow-mode evaluation, rigorous multi-site validation under time-aware admissibility, explicit failure-mode criteria, and interventional trials against standard care; with these safeguards as standard, multimodal systems can credibly improve reproducibility, timeliness of integrated diagnosis, and the safety of decision support at scale, therefore improving neuro-oncology practice in glioblastoma as well as other cancers of the central nervous system.
